# Effects of oral calcium on reproduction and postpartum health in cattle: a meta-analysis and quality assessment

**DOI:** 10.3389/fvets.2024.1357640

**Published:** 2024-04-10

**Authors:** Zheng-Ren Ma, Ling-Li Ma, Fei Zhao, Yan Bo

**Affiliations:** ^1^Linxia Animal Husbandry Technology Extension Station, Linxia, China; ^2^Linxia Animal Quarantine Station, Linxia, China; ^3^Key Laboratory of Environmental Ecology and Population Health in Northwest Minority Areas, Medicine of Northwest Minzu University, Lanzhou, China

**Keywords:** oral calcium, cattle, clinical hypocalcemia, meta-analysis, quality assessment

## Abstract

Postpartum blood calcium (Ca) concentration is related to the reproduction and health of cattle. Oral calcium supplements were given to dairy cows after calving to increase blood Ca concentration and reduce the risk of hypocalcemia. However, studies have shown that oral Ca has different effects in preventing disease. The purposes of this study were (i) to conduct a meta-analysis to evaluate the expected effect of oral Ca on incidence of calving-related diseases, pregnancy risk and milk yield in dairy cows, and (ii) to make a quality assessment of these related studies. In total, 22 eligible studies were included in this review. Meta-analysis showed that oral Ca could significantly reduce the incidence of hypocalcemia (clinical hypocalcemia: relative risk (RR) = 0.67, 95% confidence interval (CI) = [0.52, 0.87]; subclinical hypocalcemia: RR = 0.81, CI = [0.72, 0.91]), and incidence of retained placenta (RR = 0.77, CI = [0.62, 0.95]), improved blood Ca concentrations: mean difference (MD) = 0.08; 95% CI = [0.04, 0.11]. For other results, the meta-analysis revealed a lack of evidence of the correlation between oral Ca and serum magnesium (Mg) / phosphorus (P) concentration (Mg: MD = −0.04; 95% CI = [−0.10, 0.02]; P: MD = 0.05; 95% CI = [−0.10, 0.21]) or incidence of other calving-related disorders (metritis: RR = 1.06, CI = [0.94, 1.19]; ketosis: RR = 1.04, CI = [0.91, 1.18]; mastitis: RR = 1.02, CI = [0.86, 1.21]; displacement of the abomasum: RR = 0.81, CI = [0.57, 1.16]) or pregnancy risk (pregnancy risk at first service: RR = 0.99, CI = [0.94, 1.05]; overall pregnancy rate: RR = 1.03, CI = [0.98, 1.08]) or milk yield (MD = 0.44; 95% CI = [−0.24, 1.13]). The distribution of the funnel plot formed by the included studies was symmetrical, and the Egger’s test had a *p* > 0.05, indicating that there was no significant publication bias. Sensitivity analyses results suggested that the results of meta-analysis are robust. Quality assessment of the included studies revealed that the risk of bias was focused on selection bias, performance bias, detection bias and other sources of bias, and the future research should focus on these aspects.

## Introduction

Clinical hypocalcemia (also known as milk fever or parturient paresis) and subclinical hypocalcemia are common perinatal health disorders in dairy cows, which can lead to other clinical diseases or death (1–3). Postpartum milk production of dairy cows will rapidly consume circulating calcium, leading to a decrease in blood calcium (Ca) concentration (4, 5). When the total blood calcium <1.4 mmol/L, or serum total Ca < 2.0 mmol/L with clinical signs, it is diagnosed clinical hypocalcemia (6, 7). There are no obvious clinical symptoms in subclinical hypocalcemia. In published studies, subclinical hypocalcemia was defined using thresholds that varied from 1.88 to 2.35 mmol/L (8).

Ca metabolism is interrelated with other minerals. When the blood calcium concentration decreases, the blood magnesium (Mg) concentration initially increases and then decreases until it stabilizes 3–4 weeks postpartum (9, 10). Some studies have determined that found that prenatal feeding with P-deficient diet had a positive effect on Ca homeostasis of perinatal dairy cows (11, 12). In feedstuffs, sodium (Na), potassium (K), chlorine (Cl) and sulfur (S) are ionic variables in the dietary cation-anion differences (DCAD) equation, and controlling their content in the diet is the key to reducing hypocalcemia in cows (13, 14). Milk fever have effects on decreased reproductive performance, and increased prevalence of other diseases during early lactation (6, 15, 16). Ducusin et al. pointed out that the hypocalcemic condition of parturient paretic cows *in vivo* causes decreased phagocytosis and resting [Ca^2+^]_i_ in polymorphonuclear leukocytes, which may partly contribute to greater susceptibility to infection (17). Milk fever can reduce tension and contractility of the uterine muscles, leading to dystocia or retained fetal membranes (18). Although study have pointed out that the occurrence of displaced abomasum was 3.7 times more likely in cows that had subclinical hypocalcemia than in cows with normocalcemia (19), Zurr et al. have found that calcium has negative effects on abomasal motility only at extremely low levels (20). Research shows that postpartum cows will experience three different forms of subclinical hypocalcemia: transient, persistent or delayed, of which persistent and delayed hypocalcemia have harmful effects on health and milk production (5).

Postpartum Ca supplementation is performed by oral, intravenous injection and subcutaneous injection. Owing to convenience, Ca is often orally administered to dairy cows after calving to increase their postpartum blood calcium concentration and reduce the incidence of hypocalcemia (21–23). At present, most farms use the commercial product Ca bolus as a supplement, and its main components are CaCl_2_ and CaSO_4_. In addition, there are other forms of calcium supplements, such as Ca gel, Ca tubes and Ca fluid preparation (8, 24–31). Although Valldecabres et al. conducted a meta-analysis, it was revealed that oral Ca supplements were not associated with milk yield or pregnancy rate at first service (32). However, the meta-analysis included a small number of outcome indicators and did not evaluate the quality of the included studies (32). The purposes of this study were (i) to conduct a meta-analysis to evaluate the effectiveness of oral Ca on improving calving-related diseases (hypocalcemia, retained placenta, metritis, ketosis, mastitis, displacement of the abomasum), pregnancy risk and milk yield in dairy cows, and (ii) to make a quality assessment of these related studies, so as to provide references for scientific design and implementation of animal research in the future.

## Materials and methods

### Search strategies

Publications from its establishment to October 19, 2023 were searched in PubMed, Embase and Web of Science databases, and the search was not limited by language. In addition, reference tracks (Federal Bureau of Agriculture), abstracts,^1^ and Google Scholar^2^ were used to supplement the search. The search strategies used were: (hypocalcemia OR hypocalcemias OR parturient paresis OR parturient pareses OR animal milk fever) AND (cattle OR *Bos indicus* OR zebu OR zebus OR *Bos taurus* OR domestic cow OR domestic cows OR *Bos grunniens* OR yak OR yaks) AND (calcium OR calcium 40).

### Study selection criteria

According to the PRISMA guidelines for Systematic Reviews and Meta-Analysis (33), following the principles of PICO (Problem/Patient/Population, Intervention/Indicator, Comparison, Outcome), the inclusion standards are pre-defined as follows: (i) question: healthy postpartum cows; (ii) intervention: oral calcium; (iii) comparison: standard interventions except for oral calcium; (iv) results: production-related diseases and blood indicators, including clinical and subclinical hypocalcemia, and Ca, Mg, and P concentrations in the blood, pregnancy risk and milk yield; and (v) study design: control *in vivo* experiment. In contrast, the exclusion criteria were as follows: (i) there are no restrictions on specific cow breed, parity; (ii) no limit to the sources, doses, forms and frequency of oral calcium; (iii) case reports, review articles, and letters; (iv) unreliable or incomplete data.

### Data extraction

Two authors collected the details of included studies independently, and a third person adjudicated when they disagreed. These details include: (i) first author name and year of publication; (ii) characteristics of animal models (breed, parity, milk production and feed); (iii) information on treatment/control group, including the treatment drug and control, sample size, calcium form, dosage, time, and number of administrations; and (iv) outcome indicators (incidence of clinical and subclinical hypocalcemia, blood Ca concentrations; Mg and P serum concentrations; the incidence of ketosis, mastitis, metritis, displacement of the abomasum, retained placenta; pregnancy risk at first service, overall pregnancy rate and milk yield). If there are multiple groups of experimental group data with the same intervention in the literature, each group will be considered independent data and included in the analysis. In addition, the data in the graph were measured by using a digital ruler software (Getdata Graph Digitizer, version 2.25, Russia).

### Quality evaluation of included studies

The Cochrane Collaboration developed the RoB tool to assess the quality of Randomized Controlled Trial (RCT) to avoid the risk of bias in animal experiments, and the SYRCLE RoB tool is an adapted version of the Cochrane RoB tool. The SYstematic Review Center for Laboratory Animal Experimentation (SYRCLE) RoB tool was used to assess the risk of bias in animal studies by using the 10 items: sequence generation (selection bias), baseline characteristics (selection bias), allocation concealment (selection bias), random housing (performance bias), blinding (performance bias), random outcome assessment (detection bias), blinding (detection bias), incomplete outcome data (attrition bias), selective outcome reporting (reporting bias), other sources of bias (other). The evaluation results of each project classify the research into high risk, low risk or unclear risk according to PRISMA guidelines. In the result chart, red, green and yellow represent “high risk,” “low risk” and “unclear risk” respectively (34).

### Statistical analysis

This study, used RStudio software (version 5701.9.1.0) for data analysis. The mean difference (MD) with 95% confidence interval (CI) for continuous variables and relative risk (RR) with 95% CI for dichotomous variables were calculated. Heterogeneity was assessed using I^2^ and Π^2^ statistical tests, and heterogeneity was considered high, if I^2^ was greater than 50%. Sensitivity analyses were performed for outcome measures with moderate heterogeneity (heterogeneity I^2^ of nearly 50%) to assess the robustness of the results. Statistical significance was set at *p* < 0.05. Publication bias assessment was performed using Egger’s test.

## Results

### Literature screening

**Figure 1:**
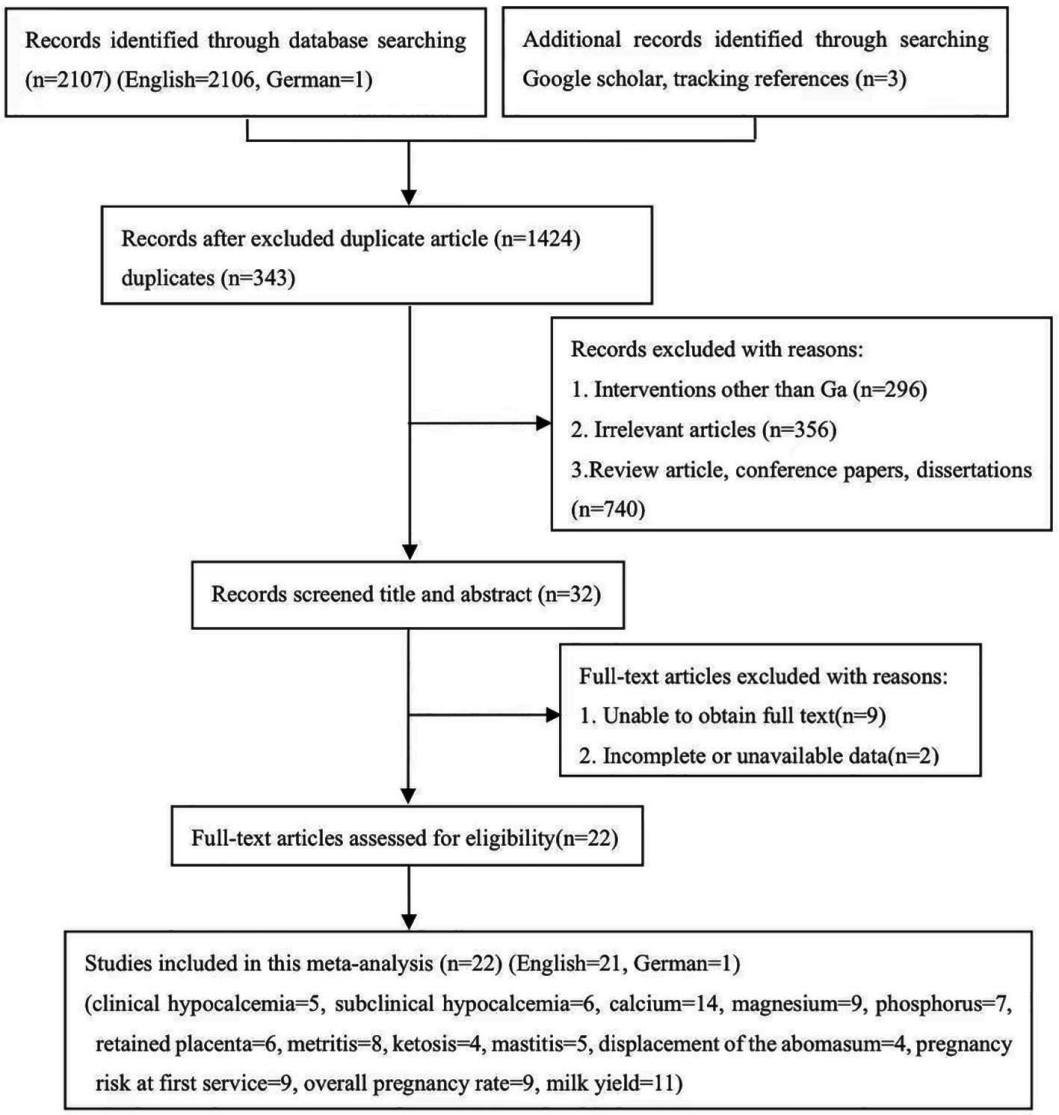
Literature screening flow chart.

A total of 2,110 potentially relevant studies were identified through a database searching and supplementary searches. 343 studies were considered duplicated, while 1,402 studies were excluded since they did not meet the inclusion criteria. Finally, 22 eligible studies were included, as shown in Figure 1 (English = 21, German = 1).

### Characteristics of the included studies

Of the 22 eligible studies, 15 studies used Holstein cows, three studies used Jersey cows, and five studies adopted crossbred cows. One study did not report the breed used for their experiment. Regarding parity, 12 studies used multiparous cows, eight used primiparous and multiparous cows, and two did not report parity. Seventeen studies reported milk production in cows, and 18 reported DCAD. The major outcome indicators were the incidence of clinical and subclinical hypocalcemia. Secondary outcome indicators include the concentration of Ca, Mg, and P in the blood; incidence of ketosis, mastitis, metritis, displacement of the abomasum, retained placenta; pregnancy risk at first service, overall pregnancy rate and milk yield. The overall characteristics of the included studies are summarized in Table 1.

**Table 1:** Characteristics of 23 included studies.

Study	Animal			Feed		Outcome
	Breed	Parity	Milk production (kg/cow/ year)	Prepartum DCAD^2^ (mEq/kg DM)	Postpartum DCAD^2^ (mEq/kg DM)	
Melendez et al. (24)	Holstein	PP and MP	10,500	−80	NR	a, f, h, i, k, l, m
Goff et al. (25)	Holstein, Jersey	MP	NR	NR	NR	b, c, f, g, i, j
Valldecabres et al. (26)	Jersey, Jersey × Holstein	MP	Herd A:8748, Herd B:9697	Herd A: −176.1 Herd B: −145.5	Herd A: 145.5 Herd B: 294	k, l, m
Kurek et al. (27)	Holstein × Friesian	3–6 years old	9,000	NR	NR	c, d, e
Hajikolaei et al. (28)	Holstein	MP	12,047	−126	339	a, g, j, l
Leno et al. (29)	Holstein	PP and MP	13,486	Herd A: −69, Herd B: −28, Herd C: −55, Herd D: −4, Herd E: 73;141, Herd F: −28	Herd A: 209, Herd B: 193, Herd C: 320, Herd D: 254, Herd E: 237, Herd F: 244	b, c, g, h, i, j, k, m
Jahani-Moghadam et al. (30)	Holstein	MP	12,993	89	373	a, b, c, d, e, f, j, k, l, m
Melendez et al. (31)	Holstein	MP	7,500	−86	196	c, d, e, m
Ramella et al. (35)	Holstein	PP and MP	9,150	33.3	188.6	b, c, d, e, h, j, m
Stevenson et al. (36)	NR	MP	NR	NR	NR	c, d, e, k, l, m
Braun et al. (37)	Swiss Brown Swiss, Simmental, Holstein × Friesian	NR	NR	NR	NR	c, d
Oetzel et al. (38)	Holstein	PP and MP	NR	Herd A: −109 Herd B: −18	Herd A: 283 Herd B: 285	f, g, h, i, k, l, m
Reitsma et al. (39)	Jersey × Holstein	PP and MP	NR	−182	262	c, g, h, l, m
Farnia et al. (40)	Holstein	MP	12,407	−113	263	c, d, e, l
Valldecabres et al. (23)	Jersey	MP	7,259	−176.1	145.5	b, c, d, e, h
Martinez et al. (41)	Holstein	PP and MP	13,635	PP: 6 ± 37 MP: −153 ± 96	PP: 394 ± 66 MP: 442 ± 38	k, l, m
Martinez et al. (42)	Holstein	PP and MP	13,635	PP: 6 ± 37 MP: −153 ± 96	PP: 394 ± 66 MP: 442 ± 38	b, c, d, h
Domino et al. (43)	Holstein	MP	12,200	−100	200	a, g, h, j, k, m
Blanc et al. (44)	Jersey × Holstein crossbreed	MP	8,235	−174	188.4	c
Jahani-Moghadam et al. (45)	Holstein	MP	10,980	−94	265	b, c, d, e
Pinedo et al. (46)	Holstein	PP and MP	9,150	−100	NR	a, c
Mahjoubi et al. (47)	Holstein	MP	12,505	−93	252	k

NR, Not reported; DCAD, Dietary cation-anion difference, DCAD (mEq/kg) = [(Na + K) − (Cl + S)]; PP, primiparous cows; MP, multiparous cows; T, Treatment; C, Control; a: Incidence of Clinical hypocalcemia; b: Incidence of Subclinical hypocalcemia; c: Ca concentrations of plasma/serum; d: Mg concentrations of serum; e: P concentrations of serum; f: Incidence of Ketosis; g: Incidence of Mastitis; h: Incidence of Metritis; i: Incidence of Displacement of the abomasum; j: Incidence of Retained placenta; k: Pregnancy risk at first service; l: Overall pregnancy rate; m, Milk yield.

The definitions of clinical diseases in the included studies are not consistent. Clinical hypocalcemia is defined as lying flat within 24 h after delivery, corrected by intravenous injection of calcium, or as the symptoms of lying weak or cold limbs without signs of physical injury within 72 h after delivery (28, 29). Subclinical hypocalcemia is defined as serum Ca ≤ 2.12 mmol/L, or blood total Ca < 2.125 mM, or ≤ 1.00 mmol/L on calving day (23, 35, 38). Ketosis is defined as a sharp drop in milk production, with acetoacetic acid in urine, or BHBA ≥1.2 mmol/L on BHBA test (24, 25, 38). Metritis is defined as foul-smelling uterine secretions that are red to brown within 14 days after delivery, or body temperature ≥ 39.5\u00B0C, lethargy, anorexia and vaginal discharge (29, 35). Displacement of the abomasum is defined as the decrease of milk production, and at the same time, when tapping and auscultating the left or right abdominal wall between the ninth and twelfth rib spaces, high-pitched tympanic resonance can be heard, with or without colic (24). Retained placenta is defined as the fetal membranes or placenta were not discharged within 24 h after delivery (28, 29, 35).

### Characteristics of the treatment

The characteristics of the interventions are presented in Table 2. Table 2 is as simple as possible, but due to the characteristics of study design or intervention, some studies have extracted multiple sets of data. The sample sizes of the included studies varied widely, ranging from 10 to 3,999 animals, with only 10 studies having the same sample size for the experimental and control groups. Accurate data of cows assigned to each experimental group could not be obtained from one study after contacting the authors (46). The treatment group cows in the included studies used different forms of Ca, including Ca gel, Ca tube, Ca bolus or Ca liquid preparation, among which Ca bolus was the most commonly used (18 studies). Cows in the control group usually have no calcium supplement, and only one study used a placebo made of test tubes filled with gelatin. There are varying many sources of Ca, and in most studies, calcium chloride (CaCl_2_) was used as the primary calcium source, one study did not report Ca sources. Eighteen studies used commercial brands of calcium. Moreover, the treatment times and frequencies in the included studies were inconsistent. In most studies, the time for oral calcium administration was 0–6, 12, and 24 h after calving. The times for the treatment regimen was 1 to 3, only two articles administer oral calcium 5 times. The dose was also inconsistent (25–110 g).

**Table 2:** Characteristics of treatment.

Study	Intervention								
	Treatment (Ca forms)	Control	Sample size(T)	Sample size(C)	Ca sources	Ca brand	Postpartum treatment time(hours)	Dose (g/Time)	Frequency of oral administration
Melendez et al. (24)	Ca gel	no oral	158	160	CaCl_2_	Super Calcium Gel®, RX Veterinary Products, Deramus, Kansas City, MO	6	60	1
Goff et al. (25)	Ca tubes	placebo^1^	25	21	Ca-propionate	calcium propionate powder, Kemin Industries, Inc., Des Moines, IA	2, 12	74	2
	Ca tubes	no oral	21	24	Ca-propionate	calcium propionate powder, Kemin Industries, Inc., Des Moines, IA	2, 12	74	2
	Ca tubes	no oral	29	28	Ca-propionate	calcium propionate powder, Kemin Industries, Inc., Des Moines, IA	2, 12	111	2
Valldecabres et al. (26)	Ca bolus^1^	no oral	364	344	CaCl_2_, Ca-propionate, Ca-lactate, CaSO_4_	QuadriCalMINI, Bio-Vet Inc., Barneveld, WI	2 h 55 min ± 2 h 10 min, 29 h 36 min ± 5 h 54 min	50 ~ 60	2
	Ca bolus^1^	no oral	230	237	CaCl_2_, Ca-propionate, Ca-lactate, CaSO_4_	QuadriCalMINI, Bio-Vet Inc., Barneveld, WI	3 h 26 min ± 2 h 24 min, 26 h 9 min ± 2 h 12 min	50 ~ 60	2
Kurek et al. (27)	Ca fluid preparation	no oral	20	20	CaCl_2_	Ionized Ca	directly before parturition, 24, 48	62.5	3
	Ca bolus^2^	no oral	20	20	CaCl_2_, CaSO_4_	pure Ca	0	43	1
Hajikolaei et al. (28)	Ca bolus	no oral	79	80	CaCl_2_	NR	0, 12	50	2
Leno et al. (29)	Cal bolus^1^	no oral	2001	1998	CaCl_2_, CaSO_4_, Ca-propionate, Ca-lactate	Quadrical, Bio-Vet Inc., Barneveld, WI	24	54 ~ 64	3
Jahani-Moghadam et al. (30)	Ca bolus	no oral	33	33	CaCl_2_, Ca-propionate, Ca-fomate	CalciZA, Pazhuhesh Parvar Zayand Co., Isfahan, Iran	0, 24	45	2
Melendez et al. (31)	Ca bolus	no oral	30	30	CaCl_2_	CalMate®, Drench-Mate, B & B Manufacturing, Sumas, WA, USA	0, 24	44	2
Ramella et al. (35)	Ca fluid preparation	no oral	60	60	Ca-formate	Calfon® oral; Bayer Saúde Animal; Brazil	5, 24	50	2
Stevenson et al. (36)	Ca fluid preparation	placebo	179	177	CaCl_2_	CaCl_2_ in oil	6 ~ 24, 19 ~ 36	49	2
Braun et al. (37)	Ca bolus	no oral	5	5	Ca-lactate	Propeller Calcium Drink®, Provet AG, 3421 Lyssach	1	80	1
Oetzel et al. (38)	Ca bolus	no oral	431	496	CaCl_2_, CaSO_4_	Bovikalc, Boehringer Ingelheim, St. Joseph, MO	0, 8 ~ 35	43	2
Reitsma et al. (39)	Ca bolus	no oral	41	42	CaCl_2_, CaSO_4_, Ca-propionate	MB Nutritional Sciences in Lubbock, Texas	24, after 24	50	2
	Ca bolus	no oral	21	22	CaCl_2_, CaSO_4_, Ca-propionate	MB Nutritional Sciences in Lubbock, Texas	24, after 24	25	2
	Ca bolus	no oral	21	22	CaCl_2_, CaSO_4_, Ca-propionate	MB Nutritional Sciences in Lubbock, Texas	24, after 24	50	2
Farnia et al. (40)	Ca fluid preparation	no oral	14	14	CaCl_2_	CaCl2 diluted in a minimal amount of water	1, 12	50	2
Valldecabres et al. (23)	Ca bolus	no oral	100	105	CaCl_2_, Ca propionate, Ca lactate, CaSO_4_	QuadriCalMINI, Bio-Vet Inc., Barneveld, WI	2 h 50 min ±2 h 01 min, 18 h 37 min ±6 h 8 min	50–60	2
Martinez et al. (41)	Ca bolus	no oral	150	150	CaCl_2_, CaSO_4_·0.5H_2_O, CaSO_4_·2H_2_O	Bovikalc; Boehringer Ingelheim Vetmedica Inc., St. Joseph, MO	d 0 and 1	86	2
	Ca bolus	no oral	150	150	CaCl_2_, CaSO_4_·0.5H_2_O, CaSO_4_·2H_2_O	Bovikalc; Boehringer Ingelheim Vetmedica Inc., St. Joseph, MO	d 0 and 1 (86 g/d), d 2, 3 and 4 (43 g/d)	86,43	5
Martinez et al. (42)	Ca bolus	no oral	6	6	CaCl_2_, CaSO_4_·0.5H_2_O, CaSO_4_·2H_2_O	Bovikalc; Boehringer Ingelheim Vetmedica Inc., St. Joseph, MO	d 0	43	1
	Ca bolus	no oral	6	6	CaCl_2_, CaSO_4_·0.5H_2_O, CaSO_4_·2H_2_O	Bovikalc; Boehringer Ingelheim Vetmedica Inc., St. Joseph, MO	d 0	86	1
	Ca bolus	no oral	150	150	CaCl_2_, CaSO_4_·0.5H_2_O, CaSO_4_·2H_2_O	Bovikalc; Boehringer Ingelheim Vetmedica Inc., St. Joseph, MO	0,24	86	2
	Ca bolus	no oral	150	150	CaCl_2_, CaSO_4_·0.5H_2_O, CaSO_4_·2H_2_O	Bovikalc; Boehringer Ingelheim Vetmedica Inc., St. Joseph, MO	d 0 and 1 (86 g/d), d 2, 3 and 4 (43 g/d)	86,43	5
Domino et al. (43)	Ca bolus	no oral	475	523	NR	Bovikalc, Boehringer Ingelheim, St. Joseph, MO	0.5, 7 ~ 32	43	2
Blanc et al. (44)	Ca bolus	no oral	11	11	CaCl_2_, CaSO_4_	Bovikalc bolus, Boehringer Ingelheim, St. Joseph, MO	1 ~ 5, 13 ~ 17	43	2
Jahani-Moghadam et al. (45)	Ca bolus	no oral	24		CaCl_2_, Ca propionate, Ca fumarate	Pajohesh Parvare Zayand Company, Isfahan, Iran	0, 24	45	2
Pinedo et al. (46)	Ca bolus	no oral	45	43	CaCl_2_, CaSO_4_	Bovikalc bolus, Boehringer Ingelheim, St. Joseph, MO	d 0,1 and 2	43	3
Mahjoubi et al. (47)	Ca bolus	no oral	115	109	CaCl_2_, CaSO_4_, calcium propionate	CALCI-UP; Ati Saman Fidar Parsa Company, Qom, Iran	3 h, 24 h ± 1 h	43	2
	Ca bolus	no oral	113	109	CaCl_2_, CaSO_4_, calcium propionate	CALCI-UP; Ati Saman Fidar Parsa Company, Qom, Iran	24 h ± 1 h, 48 h ± 1 h	43	2

Some studies have yielded multiple sets of data due to the nature of the study design or intervention. T, Treatment; C, Control; d, day; NR, Not reported; Ca tubes, The tube is filled with a paste of calcium propionate mixed with distilled water; Ca bolus^1^, Boluses also contained an undisclosed amount of niacin and vitamin D3 except Ca; Ca bolus^2^, An intraruminal bolus containing 43 g of pure calcium; Placebo^1^, A test tube filled with gelatin (Knox Gelatine, Inc., Englewood Cliffs, NJ) is made into a placebo.

### Quality evaluation of the included studies

**Figure 2:**
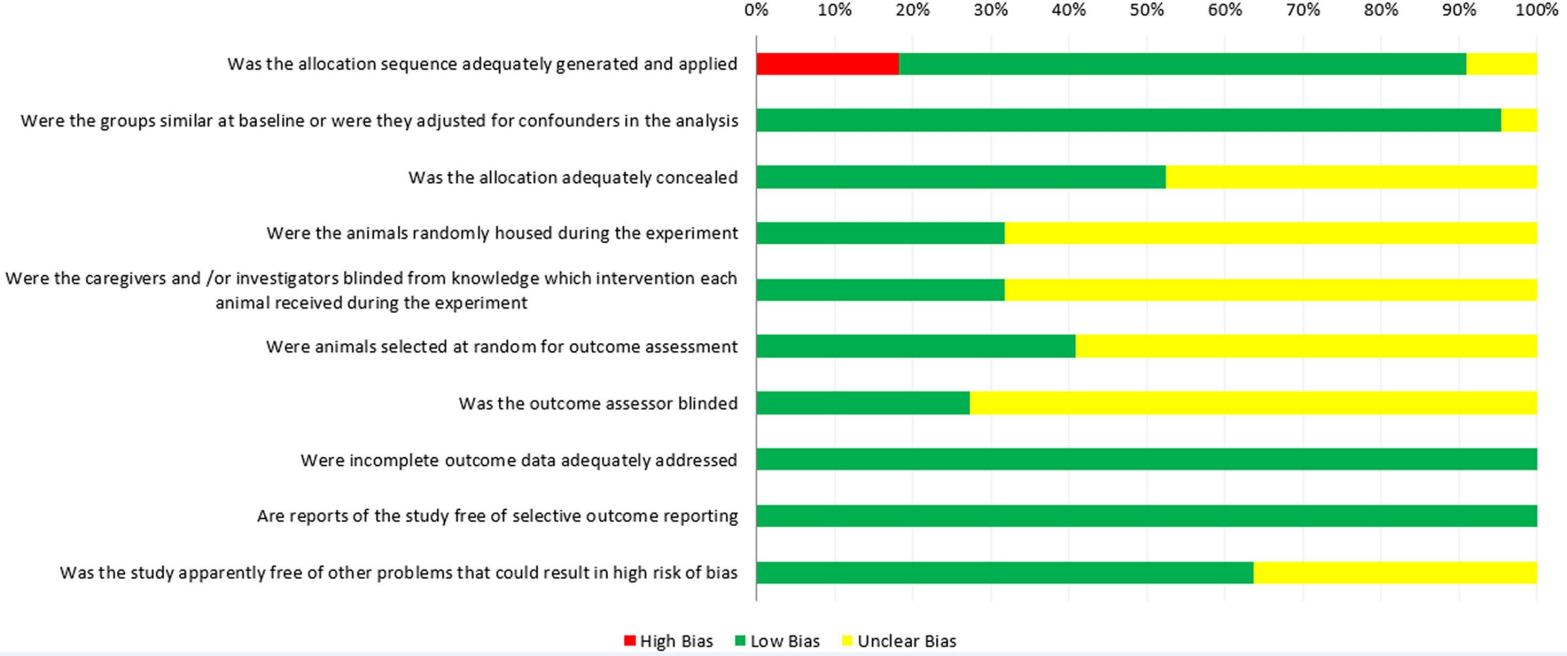
Risk of bias of the included studies by SYRCLE’s RoB tool.

The quality assessment of the included studies using SYRCLE’s RoB tool is show in Figure 2, in which all 22 studies were considered as controlled studies. Sixteen studies used random methods to generate sequences (low risk, green), however in four studies, cows were alternately assigned to treatment and control groups, based on the calving date or lactation order (high risk, red). Two studies did not describe the grouping method (unclear risk, yellow). The number of animals in the experimental and control groups in the 12 studies was also inconsistent; therefore, the risk of bias assessment of their “baseline characteristics” was unclear (unclear risk, yellow), and 19 studies adjusted for confounders in the analysis (low risk, green). Eleven studies described the allocation concealment method (low risk, green), and during the experiment implementation stage, seven studies mentioned random housing of the animals (low risk, green), while, 15 studies did not describe them (unclear risk, yellow). Lighting, humidity, temperature and other housing conditions are known to affect the research results, so it is necessary to randomize the feeding of these animals to reduce the performance bias (48). Only seven studies mentioned blinding measures used to blind trial investigators, study designers, and the outcome assessors from avoiding know which intervention each cow had received (low risk, green). Moreover, nine studies mentioned random selection of animals for assessment (low risk, green). Twenty-two studies described complete data for each primary outcome (low risk, green). In the assessment of “Is the study report irrelevant to selective outcome reporting,” all studies were considered low risk of bias (low risk, green). Eight studies mentioned other problems that could have resulted in an unclear risk of bias (low risk, green).

### Incidence of clinical and subclinical hypocalcemia

**Figure 3:**
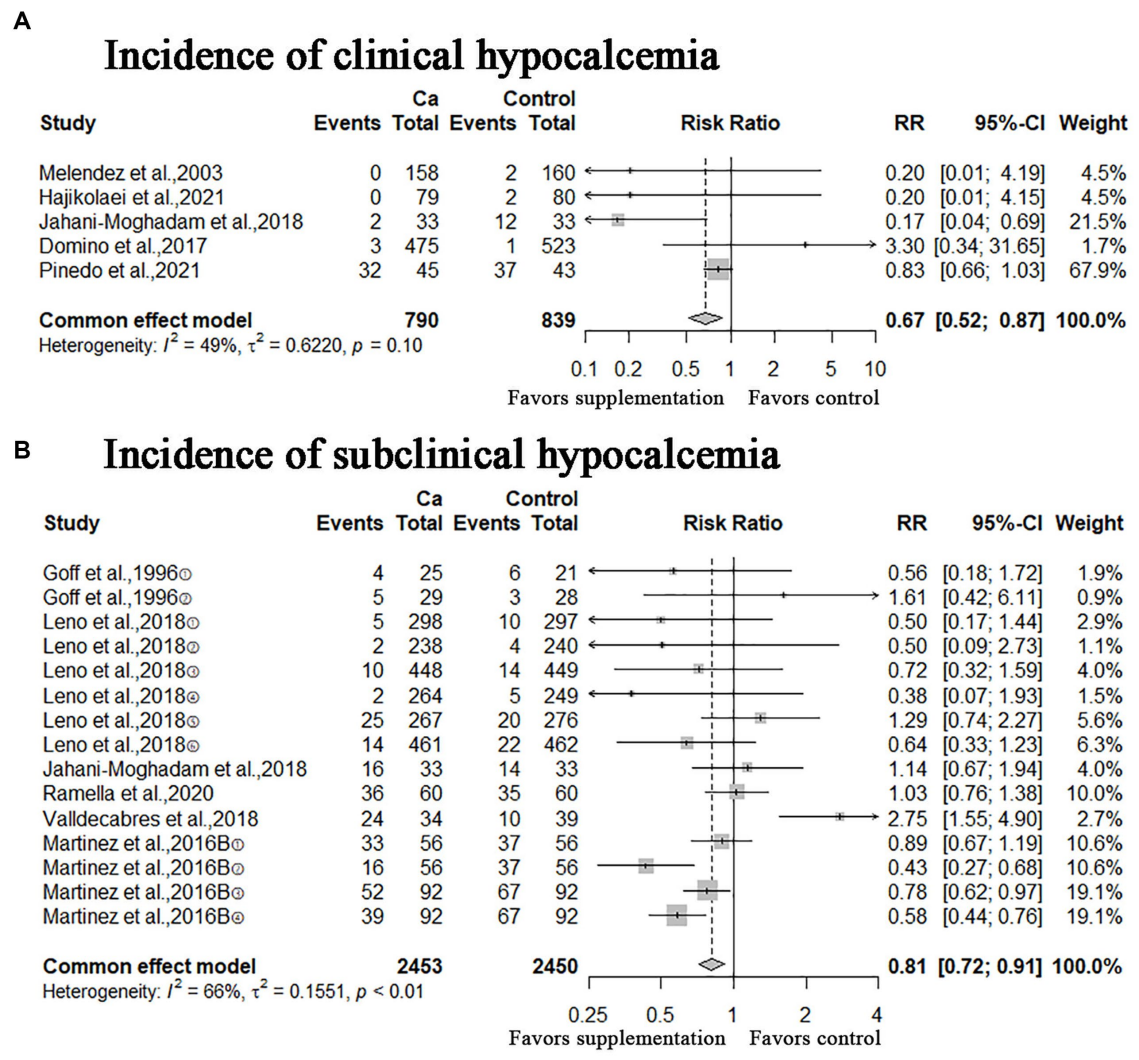
Meta-analysis of incidence of clinical hypocalcemia, subclinical hypocalcemia. Ca: Oral calcium supplements.

A meta-analysis of the five studies showed that oral Ca significantly reduced the incidence of clinical hypocalcemia, compared to the control (*n* = 1,629; RR = 0.67; 95% CI = [0.52, 0.87], *p* = 0.003; heterogeneity I^2^ = 49%; Figure 3A), and the difference between the two groups was statistically significant. The meta-analysis of six studies shown that oral Ca significantly reduced the incidence of subclinical hypocalcemia (*n* = 4,903; RR = 0.81; 95% CI = [0.72, 0.91], *p* = 0.0005; heterogeneity I^2^ = 66%; Figure 3B). The difference between the two groups was statistically significant.

### Blood indicators

**Figure 4:**
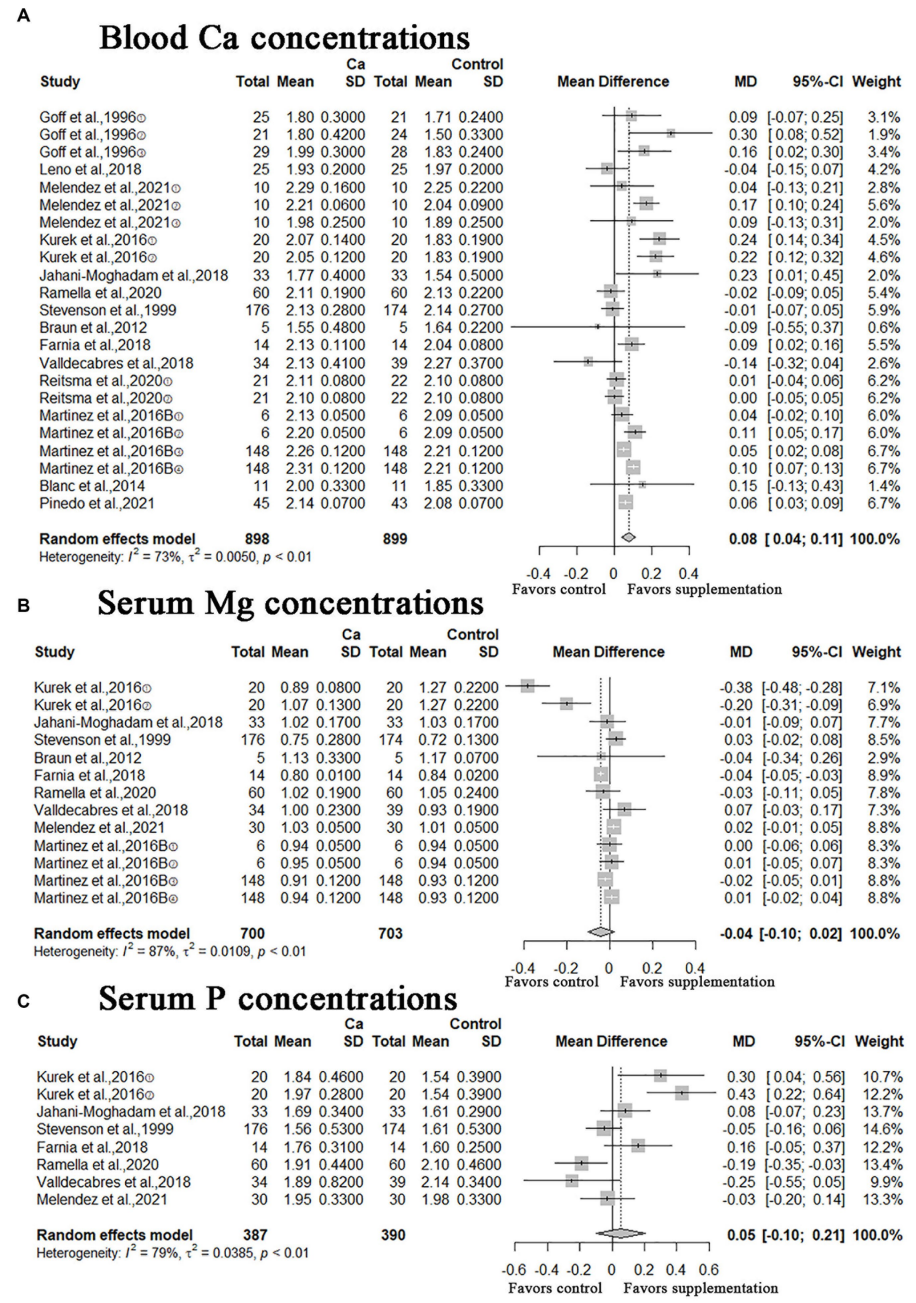
Meta-analysis of blood Ca concentrations, serum Mg and P concentrations. Ca Oral calcium supplements.

A meta-analysis of 14 studies showed that oral Ca significantly improves Ca concentration in blood compared to the control (*n* = 1797; MD = 0.08; 95% CI = [0.04, 0.11]; *p* < 0.0001; heterogeneity I^2^ = 73%; Figure 4A), the difference between the two groups was statistically significant. The meta-analysis of serum Mg and P concentrations illustrated in Figures 4B,C, the MD of serum Mg concentrations was −0.04 (95% CI = [−0.10, 0.02]; *p* = 0.2068; heterogeneity I^2^ = 87%), the result was not statistically significant; the MD of serum P concentrations was 0.05 (95% CI = [−0.10, 0.21]; *p* = 0.4944; heterogeneity I^2^ = 79%), this result was not statistically significant.

### Incidence of calving-related clinical disorders

**Figure 5:**
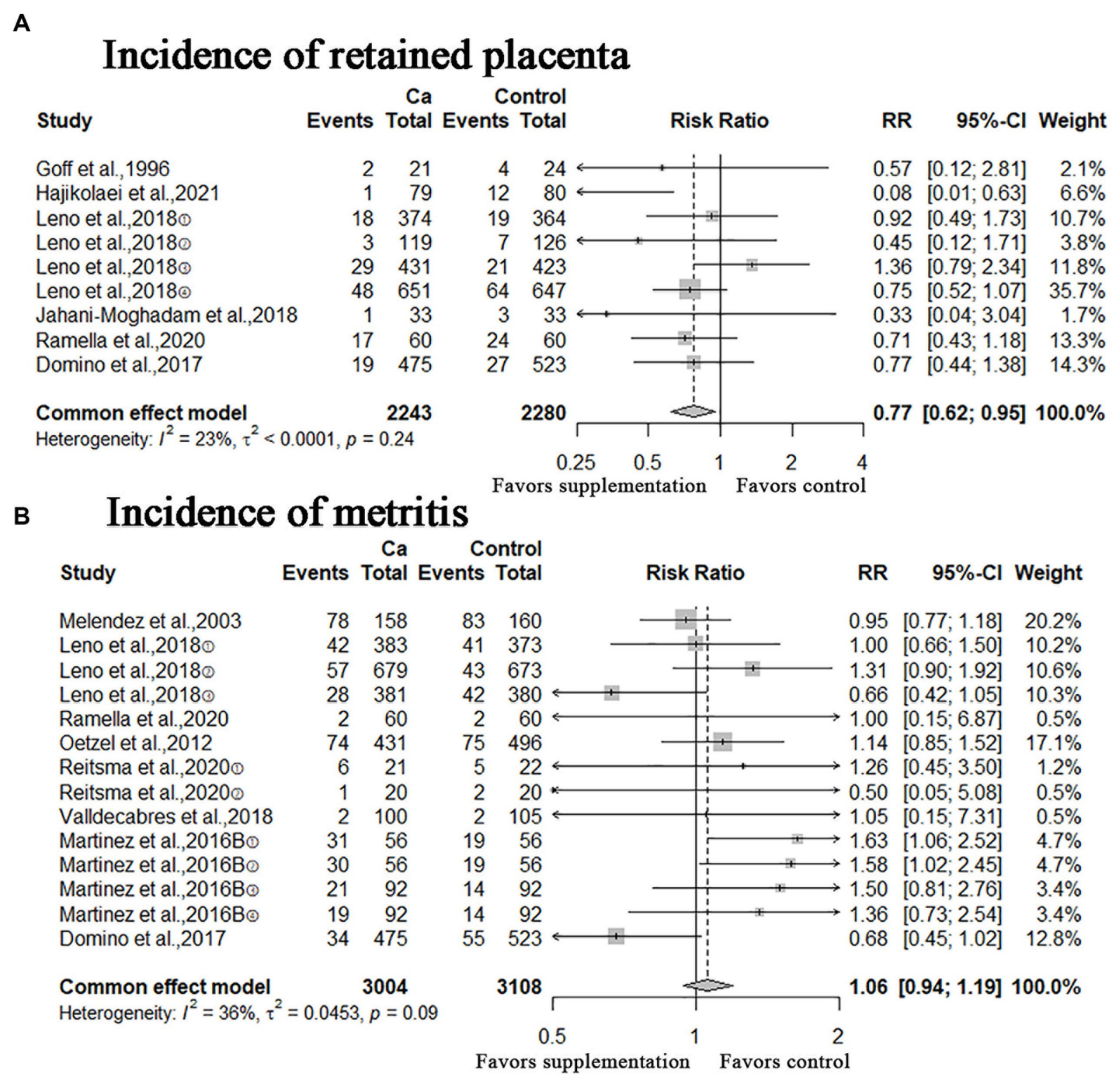
Meta-analysis of incidence of retained placenta, metritis. Ca: Oral calcium supplements.

**Figure 6:**
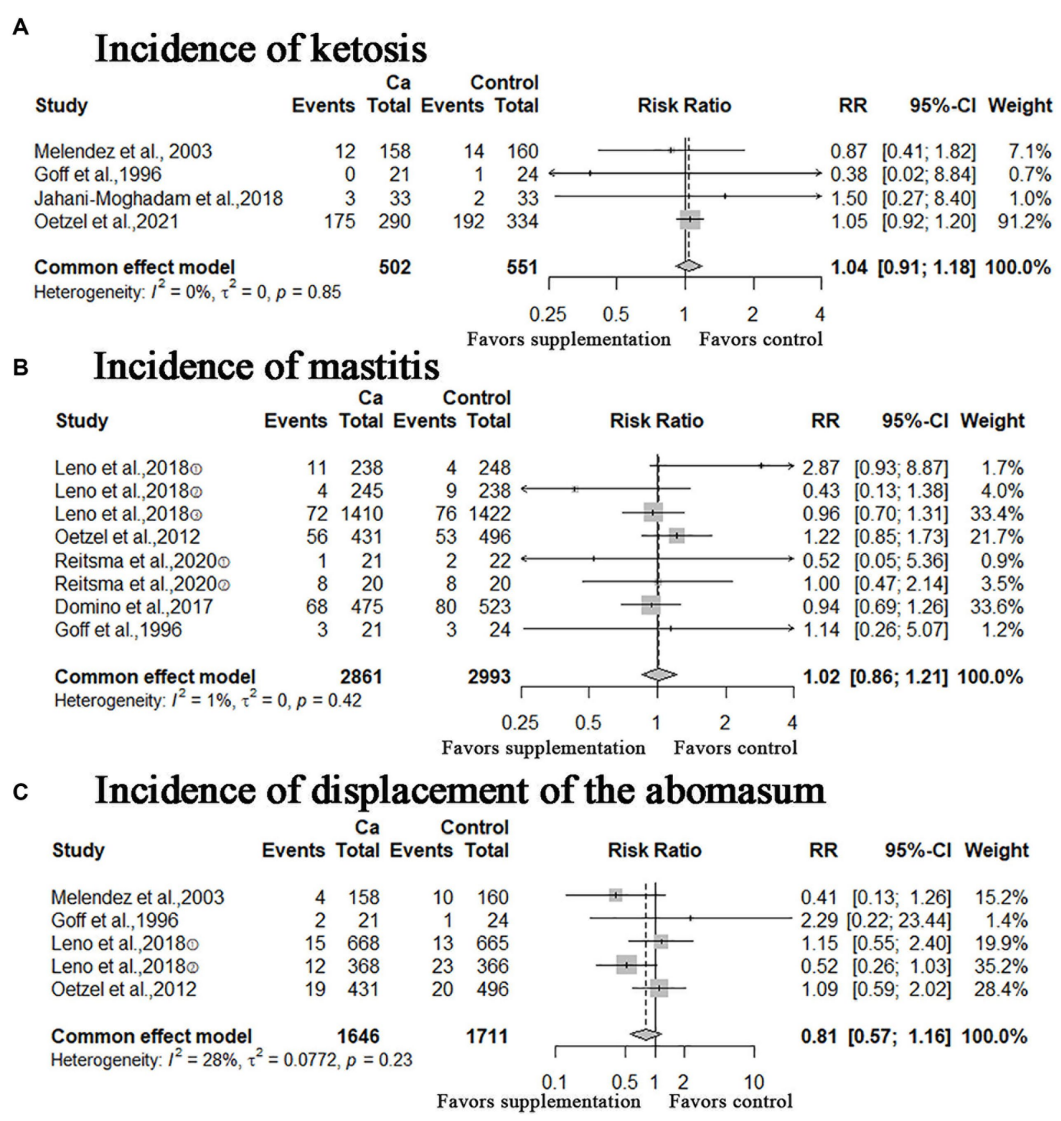
Meta-analysis of incidence of ketosis, mastitis, and displacement of the abomasum. Ca: Oral calcium supplements.

In this study, other calving-related clinical disorders including: retained placenta, metritis, ketosis, mastitis, displacement of the abomasum were included. The results of meta-analysis are shown in Figures 5, 6. A meta-analysis of the six studies showed that oral Ca significantly reduced the incidence of retained placenta, compared to the control (*n* = 4,523; RR = 0.77; 95% CI = [0.62, 0.95], *p* = 0.0151; heterogeneity I^2^ = 23%; Figure 5A), the difference between the two groups was statistically significant. The RR of incidence of metritis was 1.06 (*n* = 6,112; 95% CI = [0.94, 1.19]; *p* = 0.3567; heterogeneity I^2^ = 36%; Figure 5B); the RR of incidence of ketosis was 1.04 (*n* = 1,053, 95% CI = [0.91, 1.18]; *p* = 0.5933; heterogeneity I^2^ = 0%; Figure 6A); the RR of incidence of mastitis was 1.02 (*n* = 5,854; 95% CI = [0.86, 1.21]; *p* = 0.8436; heterogeneity I^2^ = 1%; Figure 6B); the RR of incidence of displacement of the abomasum was 0.81 (*n* = 3,357; RR = 0.81; 95% CI = [0.57, 1.16]; *p* > 0.2573; heterogeneity I^2^ = 28%; Figure 6C); however, these results are not statistically significant (incidence of metritis, ketosis, mastitis and displacement of the abomasum).

### Pregnancy risks and milk yield

**Figure 7:**
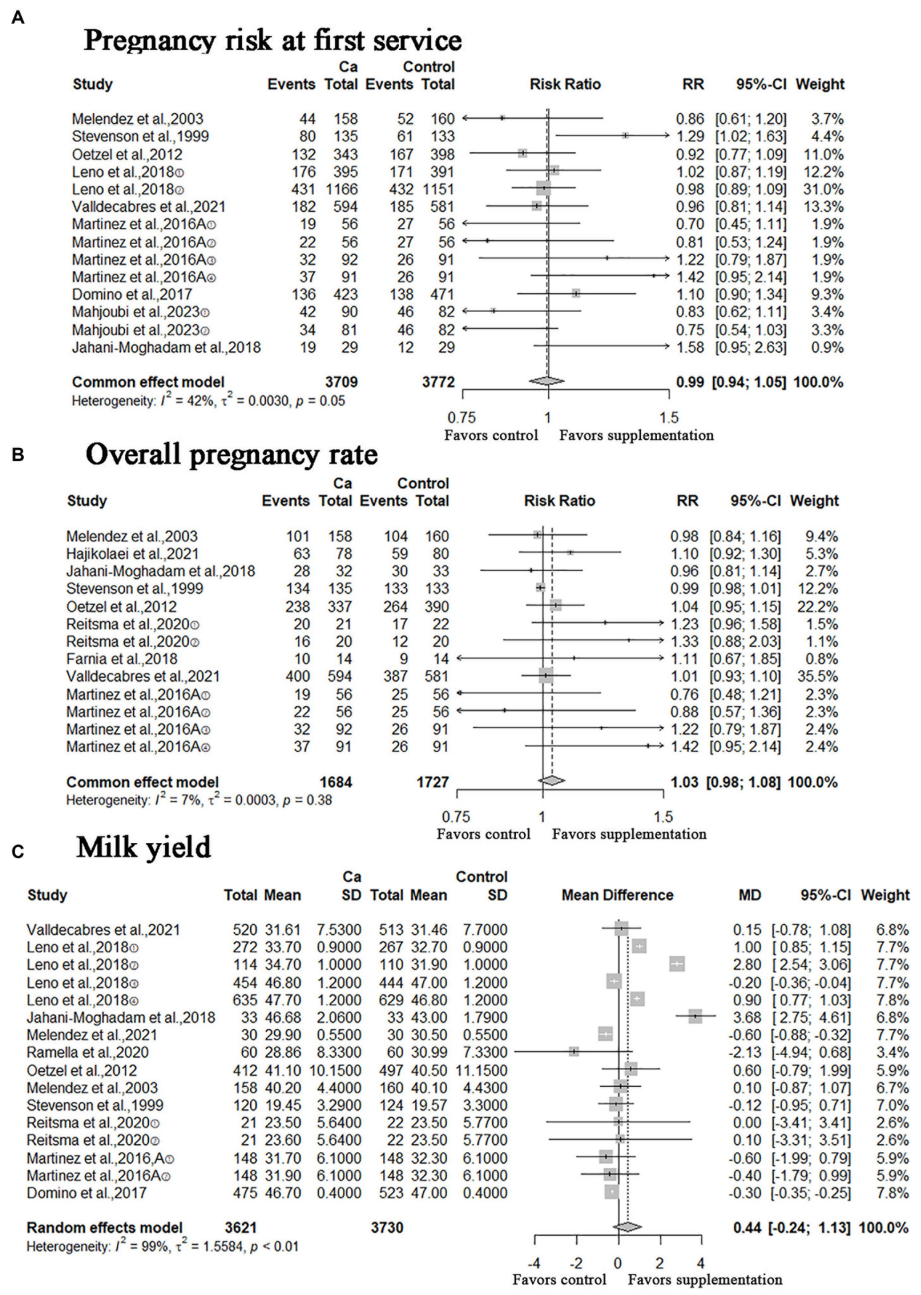
Meta-analysis of pregnancy risk at first service, overall pregnancy rate and milk yield. Ca: Oral calcium supplements.

In addition, we performed a meta-analysis of the number of pregnancies and milk yield, the RR of pregnancy risk at first service was 0.99 (*n* = 7,481; 95% CI = [0.94, 1.05]; *p* = 0.8309; heterogeneity I^2^ = 42%; Figure 7A), the result was not statistically significant; the RR of overall pregnancy rate was 1.03 (*n* = 3,411; 95% CI = [0.98, 1.08]; *p* > 0.2030; heterogeneity I^2^ = 7%; Figure 7B), this result was not statistically significant; the MD of milk yield was 0.44 (95% CI = [−0.24, 1.13]; *p* = 0.2022; heterogeneity I^2^ = 99%; Figure 7C), the result was still not statistically significant.

### Publication bias and sensitivity analyses

Publication bias was analyzed using funnel plots and Egger’s test. The distribution of the funnel plot was symmetrical (Supplementary Figure S1) the *p*-values of incidence of subclinical hypocalcemia, Ca concentration in blood, incidence of metritis, and number of pregnant cows at first service in Egger’s test were 0.8667, 0.5329, 0.7050, and 0.8959 respectively, and the results suggested that there was no publication bias between oral Ca and the incidence of these diseases. Sensitivity analyses were performed for meta-analysis of the incidence of clinical hypocalcemia, subclinical hypocalcemia and number of pregnant cows at first service to assess the robustness of the results, and the results suggested that the results of meta-analysis are robust (Supplementary Figures S2–S6).

## Discussion

Due to the increase in the cost of veterinary services and medicines and the decline in milk production, postpartum diseases of dairy cows have affected the profitability of farms (49–51). Farmers pay more and more attention to reducing the incidence of postpartum diseases in dairy cows. This meta-analysis aimed to evaluate the expected effect of oral Ca on postpartum hypocalcemia, calving-related diseases, fertility and milk yield in dairy cows. In this meta-analysis, the incidence of some calving-related diseases was significantly reduced after oral calcium administration, which include clinical hypocalcemia, subclinical hypocalcemia and retained placenta. The Ca concentrations of blood also significantly improved after Ca treatment. Clinical hypocalcemia, subclinical hypocalcemia and retained placenta are common postpartum reproductive disease. In addition, cows suffering from metabolic disorders in the early lactation period usually suffer from impaired fertility later, and may incur additional treatment costs due to delayed conception (51). Reducing the incidence of these diseases has important economic implications. Valldecabres et al. conducted a meta-analysis of the relationship between oral calcium supplements and milk production or first pregnancy rate, and found no evidence of the relationship between these outcome indicators and oral calcium (32). Our meta-analysis obtained similar results. In order to ensure the authenticity of the research evidence, we added the outcome indicators of other clinical diseases related to calving, which is more meaningful to clinical practice. However, these results were not statistically significant. Therefore, there is no evidence that oral calcium can reduce the incidence of metritis, ketosis, mastitis and abomasum displacement, or improve serum Mg and P concentrations and milk yield. According to our study selection criteria, the sources, forms or doses of oral Ca are not limited. Due to the limited number of clinical studies, it is impossible to find the relationship between various factors (Ca sources, doses and forms) and oral Ca by meta-regression, and it is also impossible to explain the influence of different calcium forms on the outcome index.

In addition, the long-term effects of oral calcium may be underestimated because cows that became sick immediately after calving (before the intervention) were removed from the herd, which may explain why some studies did not report the effect of oral calcium on the incidence of clinical hypocalcemia and its effects on blood Ca, Mg, and P concentrations. However, meta-analyses of serum Ca, Mg, P, the incidence of subclinical hypocalcemia, and milk yield were highly heterogeneous (> 50%) and remained high after sensitivity analyses. The results indicated that heterogeneity was clinical; hence we analyzed the possible sources of heterogeneity: (i) the cattle used in the included studies varied in breed, parity, and sample size. For example, some studies used Holstein cows, whereas others used crossbred cows. It is well known that the biological characteristics of dairy cows of different ages and parities (primiparous and multiparous cows) differ, inevitably affecting the experimental results; (ii) feed: prepartum and postpartum DCAD. In a meta-analysis, Santos et al., concluded that prepartum DCAD increased the Ca and P concentrations on the day of calving, as well as Ca concentrations after calving, and reduced the risk of perinatal clinical hypocalcemia, retained placenta, and metritis after calving (10, 24), therefore, diet differences could be a reason for the high heterogeneity observed in the meta-analysis results; (iii) different calcium forms, sources and brand may affect the absorption rate of animals, and thus the experimental effect; (iv) specific interventions (including interventions time, dose, and number of oral interventions) in the experimental and control groups were also not uniform; (v) the effect of confounders, such as inconsistent baselines between experimental and control groups. These differences directly affected the observed results.

The quality assessment of the results of the included studies is shown in Figure 2, the risk of bias in most animal experiments was mainly concentrated in the following aspects: (i) selection bias: cows were alternately assigned to treatment and control groups based on calving date or lactation order in four studies; (ii) performance biases: most studies did not report the random placement of cattle in the free-stall barn, and it is uncertain whether farm personnel and investigators were unaware of the interventions that each animal received during the experiment; (iii) detection biases: random selection of animals during the result evaluation process was not clearly stated; (iv) other sources of bias, inappropriate research influence by funders. Finally, the following factors should be considered in future research to avoid bias: (i) the study design should be more standardized, such as the grouping method of animals (random average grouping); (ii) the animals used in the experiments should have consistent baseline characteristics, including breed and parity; (iii) the details of the experimental method should also be consistent, including the Ca form, Ca sources, the intervention time, dose, and oral frequency; (iv) blind method is applicable to researchers, farm workers, data collectors and statistical analysts to ensure the authenticity and reliability of the results; (iv) research reports should be more detailed and provide complete data reports.

## Conclusion

This study used meta-analysis to evaluate the efficacy of oral Ca in preventing hypocalcemia or calving-related diseases, and improving pregnancy risk or milk yield in dairy cows. All analyses showed that compared with the control treatments, oral Ca administration significantly reduced the incidence of postpartum hypocalcemia (include clinical and subclinical hypocalcemia) and retained placenta, and increased blood Ca concentrations of dairy cows. However, there is no evidence that oral Ca can reduce the incidence of postpartum metritis, ketosis, mastitis and abomasum displacement, or improve serum Mg/P concentrations, pregnancy risk and milk yield. The quality evaluation of the included studies shows that the risk of bias in most animal experiments mainly center on the selection bias, performance bias, detection bias and other sources of bias. We suggest that future research should focus on these aspects and follow standardized research designs.

## Data availability statement

The original contributions presented in the study are included in the article/Supplementary material, further inquiries can be directed to the corresponding author.

## Author contributions

ZR-M: Conceptualization, Data curation, Methodology, Writing – original draft. L-LM: Data curation, Formal analysis, Writing – original draft. FZ: Conceptualization, Funding acquisition, Supervision, Writing – review & editing. YB: Writing – review & editing, Data curation, Methodology, Supervision, Conceptualization, Funding acquisition.

## Footnotes

^1^ www.ebsco.com/

^2^ www.scholar.google.com.cn

## Supplementary material

SUPPLEMENTARY Figure S1 Funnel plot of incidence of subclinical hypocalcemia, Ca concentration in plasma/serum, incidence of metritis, and number of pregnant cows at first service.

SUPPLEMENTARY Figure S2 Sensitivity analyses of incidence of clinical hypocalcemia, incidence of subclinical hypocalcemia.

SUPPLEMENTARY Figure S3 Sensitivity analyses of blood Ca concentrations, serum Mg and P concentrations.

SUPPLEMENTARY Figure S4 Sensitivity analyses of incidence of retained placenta, metritis.

SUPPLEMENTARY Figure S5 Sensitivity analyses of incidence of ketosis, mastitis, and displacement of the abomasum.

SUPPLEMENTARY Figure S6 Sensitivity analyses of pregnancy risk at first service, overall pregnancy rate and milk yield.
